# Involvement of TOB1 on autophagy in gastric cancer AGS cells *via* decreasing the activation of AKT/mTOR signaling pathway

**DOI:** 10.7717/peerj.12904

**Published:** 2022-02-04

**Authors:** Dong Wang, Yunlong Li, Shuning Sui, Mengdi Cai, Kexian Dong, Ping Wang, Xiao Liang, Songbin Fu, Jingcui Yu

**Affiliations:** 1 Scientific Research Centre, The Second Affiliated Hospital of Harbin Medical University, Harbin, China; 2 Department of General Surgery, The Second Affiliated Hospital of Harbin Medical University, Harbin, China; 3 Key Laboratory of Preservation of Human Genetic Resources and Disease Control in China, Ministry of Education, Harbin Medical University, Harbin, China

**Keywords:** Gastric cancer, *TOB1* gene, Autophagy, AKT/mTOR signaling pathway

## Abstract

**Background:**

We previously identified the tumor suppressor gene *TOB1* as related to gastric cancer. The purpose of this study was to explore whether TOB1 induces autophagy through the AKT/mTOR signaling pathway in gastric cancer.

**Methods:**

Western blotting was used to detect the protein levels of TOB1, LC3, AKT, mTOR, phosphorylated (p) AKT, and p-mTOR. A double fluorescent GFP-RFP-LC3 fusion protein was used to trace autophagy by laser confocal microscopy. Autophagosomes were observed by transmission electron microscopy.

**Results:**

The conversion of LC3-I to LC3-II and the LC3-II/LC3-I ratio were significantly increased in AGS cells overexpressing TOB1 compared with control cells. Fluorescence imaging showed LC3 puncta at 48 h, and these puncta increased significantly at 72 h after TOB1 transfection compared with control tumor cells. The presence of autophagosomes in AGS cells was observed at 72 h after TOB1 transfection by transmission electron microscopy, and no autophagosomes were found in the control cells. Moreover, the levels of p-AKT and p -mTOR were lower in AGS cells than in control cancer cells.

**Conclusion:**

Our results provide novel insight that TOB1 might suppress gastric cancer by inducing autophagy, possibly through decreasing phosphorylation and the subsequent activation of the AKT/mTOR signaling pathway.

## Introduction

Gastric cancer (GC) is the fifth most frequently diagnosed type of cancer and the third leading cause of cancer-related death worldwide (Bray et al., 2018). Challenges remain in exploring the molecular mechanism of GC development and finding effective treatments. The etiology of gastric cancer involves multiple factors, pathways and complex stages. Nonetheless, the activation of oncogenes and inactivation of tumor suppressor genes are the main molecular mechanisms of gastric cancer development.

The Transducer of ERBB2, 1 (*TOB1*) gene is a member of the TOB/BTG antiproliferative protein family. The encoded nucleoprotein can bind to transcription factors in the nucleus and play a regulatory role in transcription, thereby inhibiting cancer cell proliferation (Suzuki et al., 2002). TOB1 was reported to activate deadenylation and to be involved in the posttranscriptional regulation of gene expression (Mauxion et al., 2009). Mutations in the 3′ UTR of the *TOB1* gene decrease TOB1 expression (Rheinbay et al., 2020). Accumulating evidence suggests that the activation of TOB1 may induce autophagy in gastric cancer through the AKT pathway. Jiao et al. (2012) found that TOB1 functions as a central negative regulator of the PI3K/Akt pathway to control lung cancer occurrence and metastasis by negatively regulating EGFR expression and increasing PTEN expression. The PI3K/AKT/mTOR pathway was found to be activated in gastric cancer (Tapia et al., 2014). Moreover, the anti-gastric tumor activity of tetrandrine depended on inducing autophagy through the upregulation of autophagy-related proteins and the decreased phosphorylation of AKT/mTOR (Bai et al., 2018).

In our previous study, microsatellite markers were used to analyze the loss of heterozygosity (LOH) of chromosome 17 in 45 patients with primary gastric cancer. TOB1 was identified in the smallest missing region (17q21.33 LOH region), providing the first evidence that TOB1 is a gastric cancer-related tumor suppressor (Yu et al., 2008a; Yu et al., 2008b). Furthermore, by analyzing *TOB1* gene expression in 97 cases of primary gastric cancer and four gastric cancer cell lines, we revealed that TOB1 was downregulated in 73 gastric cancer tissues and three out of four gastric cancer cell lines, and the ratio of phosphorylated (p)-TOB1 to total TOB1 protein was increased in gastric cancer cells. An elevated ratio of p-TOB1 was associated with functional inactivation of the *TOB1* gene in gastric cancer (Yu et al., 2011). Moreover, decreased expression levels of DAL-1 and TOB1 were associated with shorter survival of gastric cancer patients (Guo et al., 2019). Therefore, this study evaluated whether and how the *TOB1* gene induces autophagy in gastric cancer, aiming to provide more evidence for TOB1 as a potential therapeutic target in gastric cancer.

## Materials and Methods

### Cell line and cell culture

The human gastric adenocarcinoma cell line AGS was purchased from the American Type Culture Collection (ATCC, Manassas, VA, USA) and was authenticated by Beijing Microread Genetics Co., Ltd. (Beijing, China) using short tandem repeat analysis. These gastric cancer cells were cultured in F-12K medium (Gibco BRL, Waltham, MA, USA) supplemented with 10% fetal bovine serum (FBS, PAA, Austria) in a humidified 5% CO_2_ atmosphere at 37 °C.

### Establishment of a gastric cancer cell line overexpressing TOB1

TOB1 overexpression lentivirus (GV358-TOB1) and lentiviral vector (GV358) were obtained from GeneChem Co., Ltd. (Shanghai, China). Gastric cancer cells were transduced according to the manufacturer’s instructions. In brief, 1 × 10^5^ cells/well in 500 μL medium were cultured overnight in a 24-well culture plate. Then, the medium was replaced with 400 μL of enhancement fluid, 50 μL of 1 × 10^8^ TU/mL virus solution and 50 μL of polybrene (50 μg/mL). The medium was replenished after 24 h, and the cells were harvested at 48 and 72 h.

### GFP-RFP-LC3 adenovirus transduction

mRFP-GFP-LC3 double-labeled adenovirus was purchased from Hanbio (Shanghai, China). Transductions were performed with 1 × 10^5^ cells/well in 500 μL medium in a 12-well culture plate overnight. The culture medium was replenished with 488 μL of enhancement solution containing 2 μL 1 × 10^10^ FPU/mL virus solution. Images were captured with a laser confocal microscope (Leica, London, UK) at 48 and 72 h.

### Western blotting

Gastric cancer cells were harvested and lysed in RIPA buffer (Applygen, C1053+, China) containing protease inhibitor (Roche, 04693159001) and phosphatase inhibitor (MCE, HY-K0022, China). The protein samples were separated by sodium dodecyl sulfate–polyacrylamide gel electrophoresis (SDS–PAGE; Beyotime Biotechnology, China) and then transferred to polyvinylidene difluoride (PVDF) membranes (Immobilon™-PSQ Membranes; Sigma–Aldrich, St. Louis, MI, USA). The membranes were blocked with 5% nonfat milk for 2 h at room temperature and then hybridized with primary antibodies against TOB1 (1:500, Abcam, ab168947), LC3B (1:2500, Abcam, ab192890), AKT (1:5000, Abcam, ab179463), mTOR (1:5000, Abcam, ab32028), p-AKT (Ser473) (1:500, Abcam, ab81283), p-mTOR (Ser2448) (1:500, Abcam, ab109268), and β-actin (1:500, ZSGB-BIO, TA-09) overnight at 4 °C, followed by incubation with secondary antibodies (anti-rabbit or anti-mouse antibodies, 1:5000, Rockland, Limerick, PA, USA). Blots were imaged using an Odyssey Infrared Imaging System (Li-COR, Lincoln, NE, USA), and band density was analyzed with ImageJ software.

### Transmission electron microscopy

Lentivirus-transfected gastric cancer cells were collected and fixed with prechilled 4% glutaraldehyde fixative (Solarbio, Beijing, China) for 24 h at 4 °C. Then, 100 μL melted 3% agarose was added, and the resulting mixture was centrifuged at 60,000 rpm for 30 s. The cell pellet was rinsed with 0.1 mol/L sodium arsonate buffer and fixed with 1% citric acid aqueous solution for 2 h. After ethanol gradient dehydration, the cells were treated with propylene oxide twice, immersed in propylene oxide and Epon812 (1:1 and 1:2 mixtures) for 1 h and then embedded, sliced, stained, and observed under a transmission electron microscope (Hitachi, Tokyo, Japan).

### Statistical analysis

All data are presented as the mean ±SD. A paired-samples t test was used to compare differences between two groups. Pearson correlation analysis was used to analyze the correlation between TOB1 and ATG3 or ATG7 in sequencing data at the RNA level. *P < 0.05, **P < 0.01, and ***P < 0.001 were considered to indicate statistical significance.

## Results

### Overexpression of TOB1 in gastric cancer cells increased the conversion of LC3-I to LC3-II

**Figure 1:**
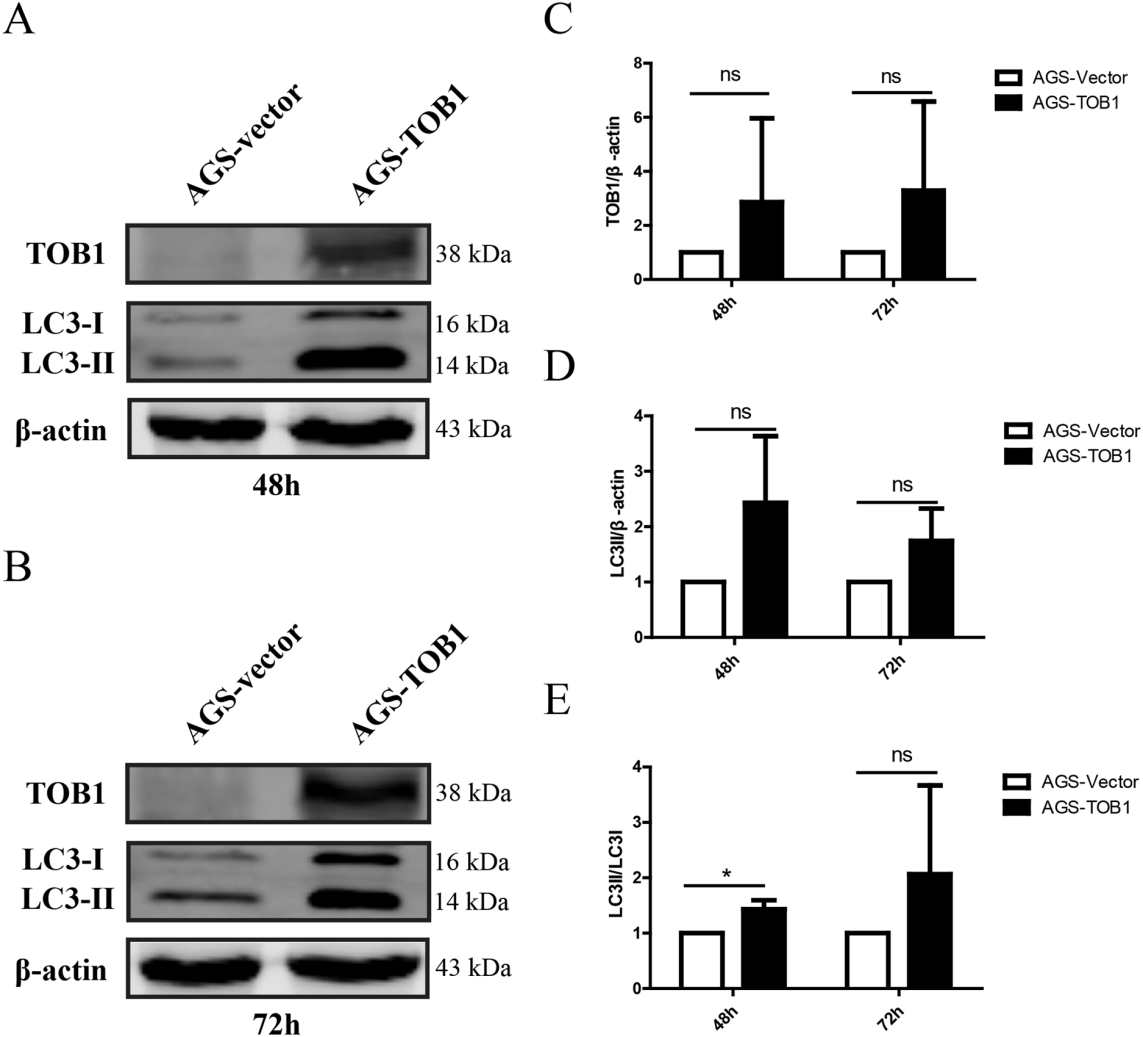
Over-expression of TOB1 increased the conversion from LC3-I to LC3-II proteins in gastric cancer cells. (A) Western blot analysis of the expression of TOB1 and LC3 proteins in AGS cells 48 h after TOB1 over-expression. (B) Western blot analysis of the expression of TOB1 and LC3 proteins in AGS cells 72 h after of TOB1 over-expression. (C) The quantification of TOB1 over-expression. (D) The quantification of LC3-II proteins in AGS cells. (E) The ratio of conversion from LC3-I to LC3-II in AGS cells (*P < 0.05).

To confirm the effect of TOB1 on autophagy in gastric cancer cells, we selected the gastric cancer cell line AGS, which expresses low levels of endogenous TOB1 protein, and transiently transduced these cells with GV358-TOB1 lentivirus or empty GV358 vector lentivirus (as a control). TOB1 protein levels were detected by western blot. As shown in Fig. 1, TOB1 protein levels in GV358-TOB1 lentivirus-transduced AGS cells were significantly enhanced compared to those in control cells at 48 and 72 h. Then, total protein was extracted after 48 and 72 h of transduction, and LC3 protein levels in AGS cells were detected by western blot (Figs. 1A–1C). The results showed that compared with control cells, cells overexpressing TOB1 showed increased LC3-II protein levels (Fig. 1D). The upward trend persisted even after 72 h, regardless of the slight increase in LC3-I protein in the tumor cells, indicating that LC3-I was largely converted to LC3-II during the formation of autophagosomes. Consistently, the conversion of LC3-I to LC3-II was more pronounced in cells with TOB1 overexpression at 48 h (*P = 0.0457; Fig. 1E) than at 72 h. This finding indicates that overexpression of the *TOB1* gene can induce autophagy in AGS gastric cancer cells.

**Figure 2:**
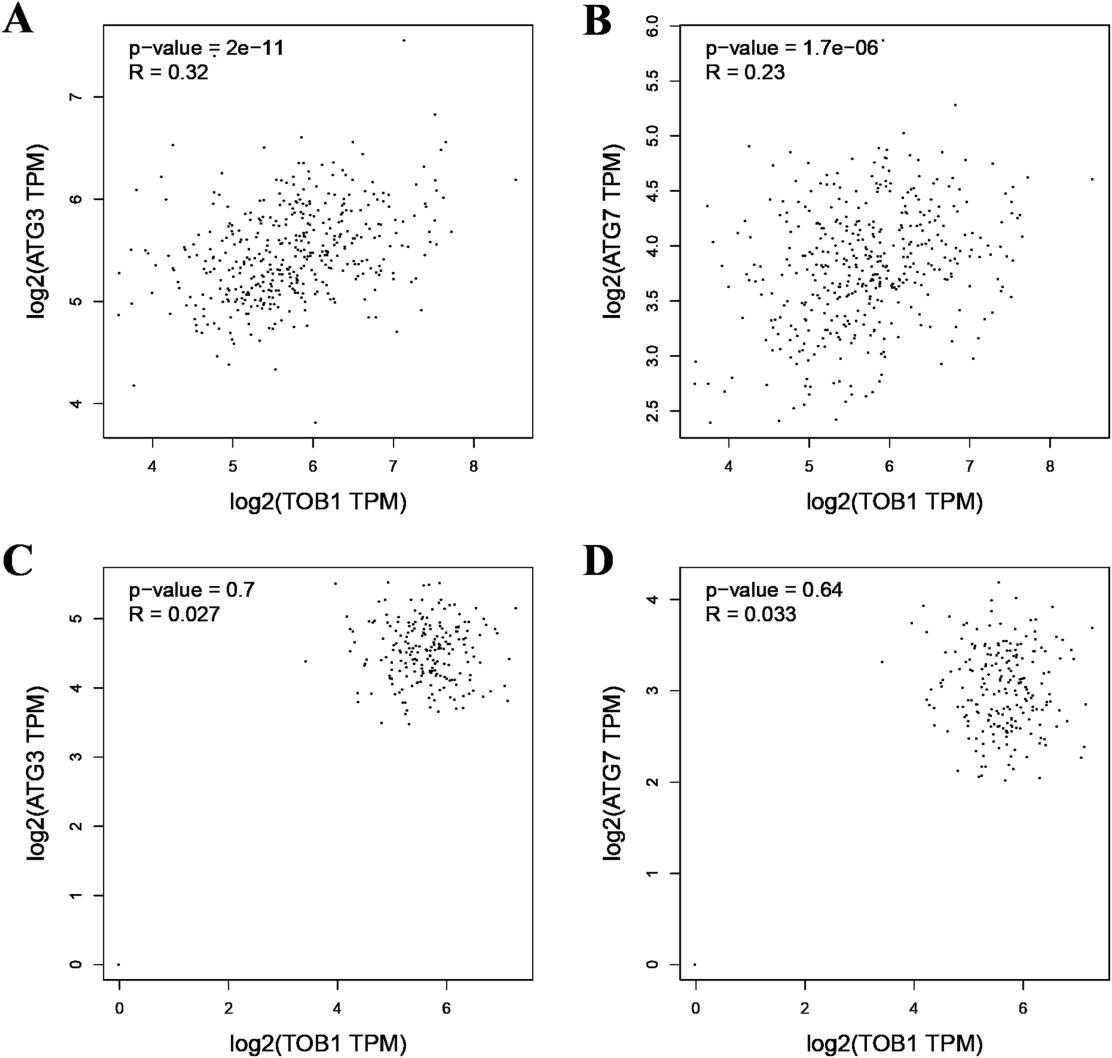
The pearson correlation analysis between TOB1 and ATG3 or ATG7 in gastric cancer tissues and normal gastric tissues. (A) The correlation analysis of TOB1 and ATG3 in gastric cancer tissues. (B) The correlation analysis of TOB1 and ATG7 in gastric cancer tissues. (C) The correlation analysis of TOB1 and ATG3 in normal gastric tissues. (D) The correlation analysis of TOB1 and ATG7 in normal gastric tissues.

As the formation of LC3-II depends on ATG3 and ATG7, pearson correlation analysis was used to analyze the correlation between TOB1 and ATG3 or ATG7 in 408 gastric cancer tissues (dataset source: TCGA) and 211 normal gastric tissues (dataset sources: TCGA, 36; and GTEx, 175) with the web server GEPIA (http://gepia2.cancer-pku.cn/#correlation). As shown in Figs. 2A and 2B, the expression level of TOB1 was positively correlated with the expression levels of ATG3 (R = 0.32, ***P = 2 × e^−11^) and ATG7 (R = 0.23, ***P = 1.7 × e^−06^) in gastric cancer tissues but not in normal gastric tissue (ATG3, R = 0.027, *P* = 0.7; ATG7, R = 0.033, *P* = 0.64) (Figs. 2C and 2D). These data provide further support that TOB1 may promote the conversion of the autophagy marker LC3-I to LC3-II in gastric cancer.

### Tracing autophagy in gastric cancer cells after the overexpression of TOB1

**Figure 3:**
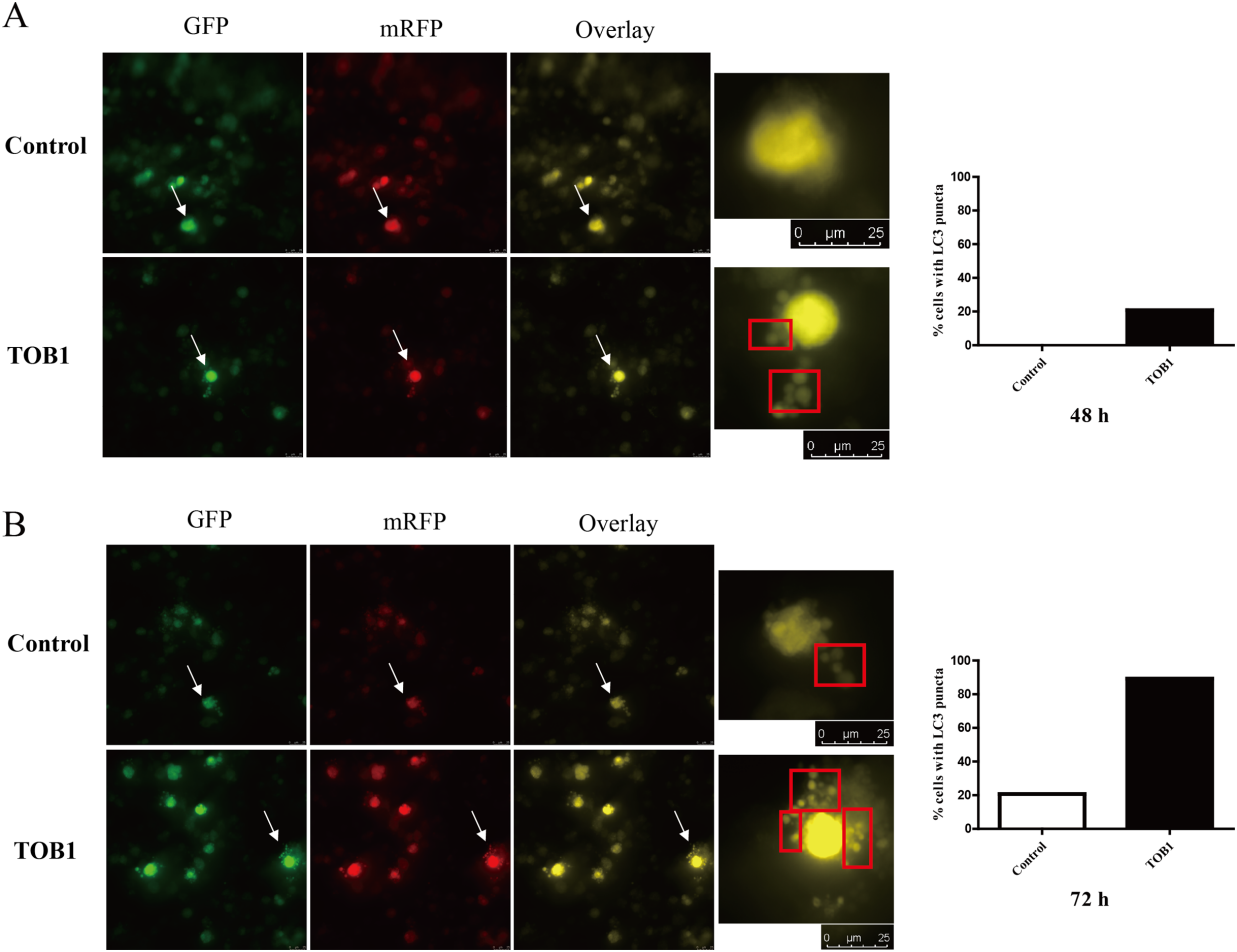
LC3 puncta formation under confocal microscope in gastric cancer cells. The white arrows indicated the target cells, cells were grouped into two categories: with LC3 puncta (red box) and without LC3 puncta. The bar charts are drawn based on the percentage of the cells scored that have five or more puncta in each group. At 48 h, there is no LC3 puncta existed in control group (20 cells) and five cells with five or more LC3 puncta in TOB1 group (24 cells), the percentage of the cells scored is 0% *vs* 20.83%. At 72 h, six cells in control group (29 cells) and 25 cells in TOB1 group (28 cells) with five or more LC3 puncta, the percentage of the cells scored is 20.69% *vs* 89.29%. (A) The LC3 puncta in AGS cells 48 h after TOB1 over-expression. (B) The LC3 puncta in AGS cells 72 h after TOB1 over-expression.

To dynamically observe the effect of TOB1 expression on autophagy in gastric cancer cells, we used a two-color fluorescent GFP-RFP-LC3 fusion protein to trace the process of autophagy. AGS cells were transduced with GFP-RFP-LC3 adenovirus and cultured for 24 h before transduction of the TOB1 lentivirus. Under normal conditions, LC3 was present in the cytoplasm. When the cells were not undergoing autophagy, the GFP (green)-RFP (red)-LC3 fusion protein showed a uniform distribution. When the cells underwent autophagy, the GFP-RFP-LC3 fusion protein translocated to the autophagosomal membrane, and the spots formed by the aggregation of red and green fluorescence were observed under a confocal microscope; the signals corresponding to LC3 fusion puncta represented autophagosomes. As shown in Fig. 3, LC3 puncta were observed in gastric cancer cells 48 h after TOB1 lentivirus transduction, and no clear LC3 puncta formed in gastric cancer cells transduced with the control lentivirus (Fig. 3A). Moreover, at 72 h, significantly more LC3 puncta were present in gastric cancer cells transduced with TOB1 lentivirus than in control cells (Fig. 3B), indicating that overexpression of the *TOB1* gene can induce autophagy in gastric cancer cells.

### Autophagosomes in gastric cancer cells overexpressing TOB1

**Figure 4:**
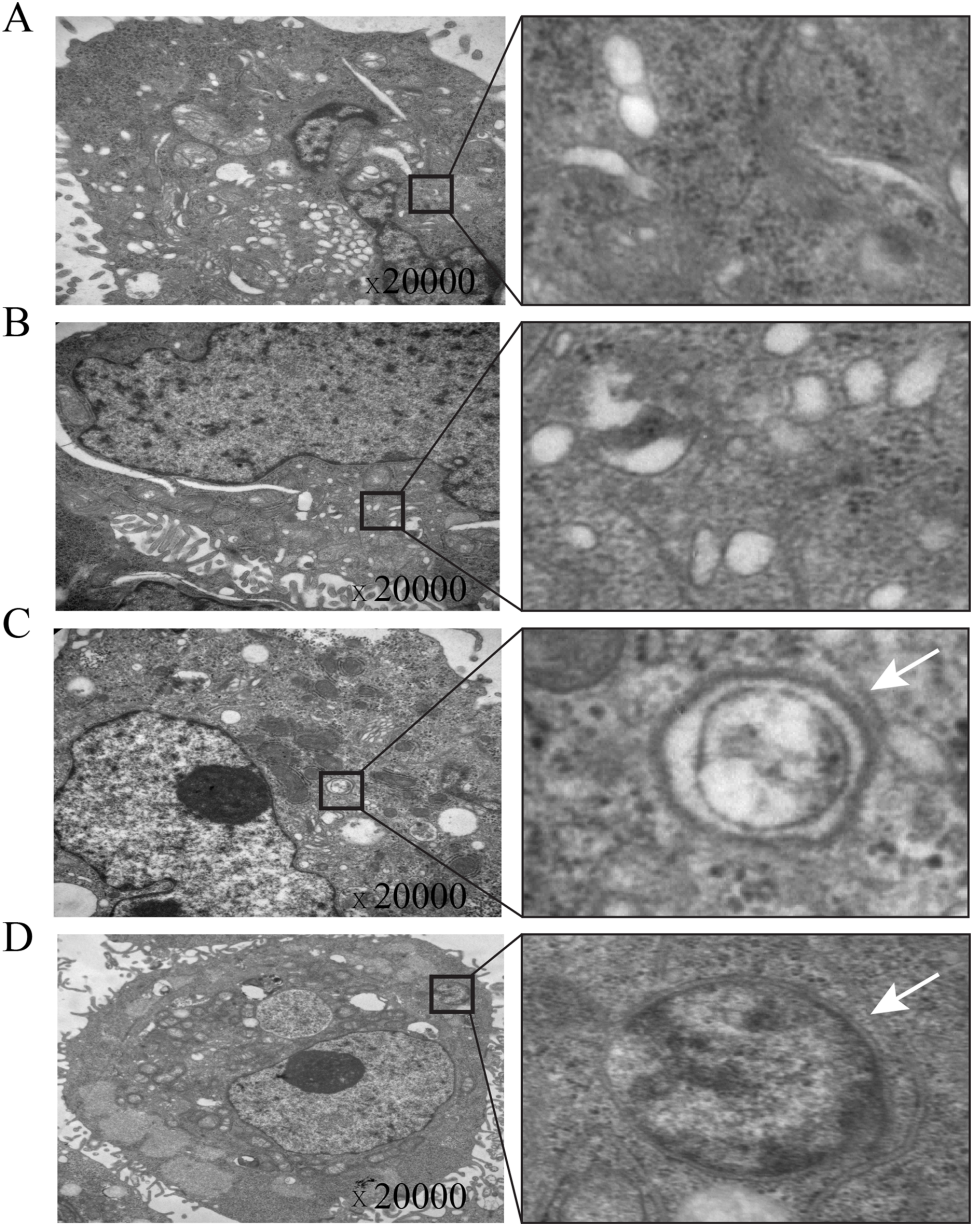
Electron microscopic imaging of intracellular autophagosomes in gastric cancer cells. (A & B) The autophagosomes were not found in control-AGS cells. (C & D) The autophagosomes were observed in TOB1-overpressed AGS cells.

Transmission electron microscopy is currently considered the gold standard for observing autophagosomes. To verify that TOB1 overexpression induces autophagosome formation, we transiently transduced the TOB1 lentivirus into AGS cells and observed the ultrastructure of these cells by transmission electron microscopy. The results showed that in TOB1 lentivirus-transduced AGS cells, typical autophagosomes appeared with bilayer membrane structures containing digested and degraded cytoplasmic components, organelles such as mitochondria, or endoplasmic reticulum fragments (arrows in Figs. 4C and 4D). However, autophagosomes were not found in AGS cells transduced with control lentivirus (Figs. 4A and 4B). These results further indicated that overexpression of the *TOB1* gene can induce autophagy in gastric cancer cells.

### TOB1 overexpression decreased the phosphorylation of AKT and mTOR in gastric cancer cells

To further investigate the mechanism underlying TOB1-induced autophagy, we detected the expression levels of AKT and mTOR, key players in the classical autophagy signaling pathway (PI3K/AKT/mTOR).

**Figure 5:**
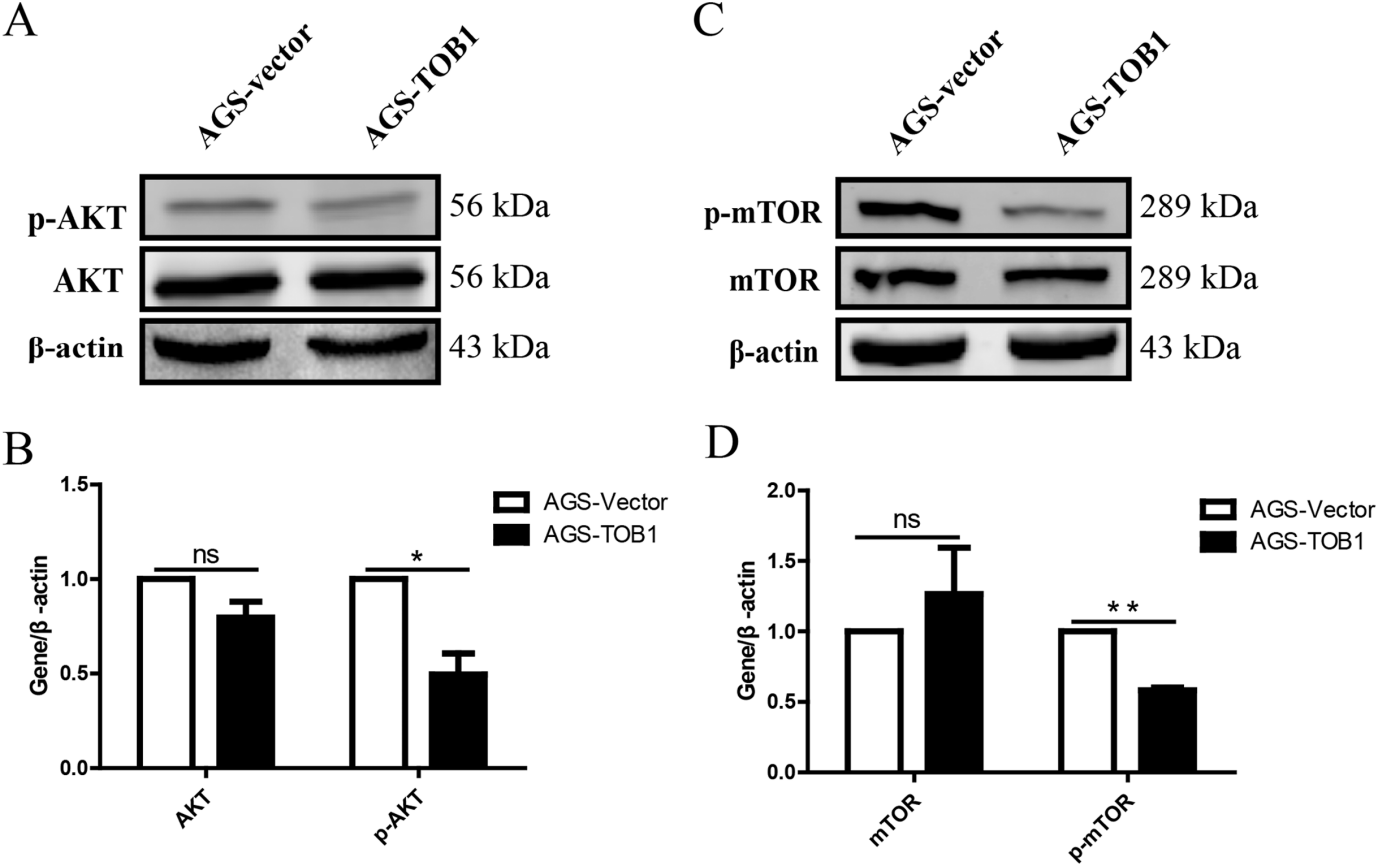
Over-expression of TOB1 induced autophagy *via* suppressing the activation of AKT and mTOR signal pathways in gastric cancer cells. (A) The level of AKT and p-AKT protein 72 h after TOB1 over-expression in AGS cells were measured by Western blot. (B) The quantification of AKT and p-AKT protein (*P < 0.05). (C) The expression of mTOR and p-mTOR protein 72 h after TOB1 over-expression in AGS cells were measured by Western blot. (D) The quantification of mTOR and p-mTOR protein level (**P < 0.01).

The results showed that p-AKT levels were significantly decreased in gastric cancer cells overexpressing TOB1 compared with control cells (Figs. 5A and 5B, *P = 0.0459), indicating that overexpression of the *TOB1* gene can inhibit AKT phosphorylative activation.

To clarify the effect of the TOB1 on the mTOR signaling pathway, western blotting was used to analyze the levels of mTOR and phosphorylated mTOR (p-mTOR) in AGS cells at 72 h after transient transduction of the TOB1 lentivirus (Fig. 5C). The data showed that compared with the control cells, the TOB1-overexpressing gastric cancer cells had a significantly reduced content of p-mTOR (Fig. 5D, **P = 0.0011), indicating that overexpression of the *TOB1* gene may inhibit the phosphorylative activation of mTOR.

## Discussion

Autophagy may play context-dependent roles in the occurrence and progression of gastric cancer; it either promotes tumor growth or contributes to tumor suppression (White & DiPaola, 2009). Both effects involve complex regulatory networks, such as those mediated by p53, PI3K/AKT/mTOR, Ras, and microRNA (Zhou et al., 2016). Our group has demonstrated that TOB1 is a tumor suppressor in gastric cancer (Guan et al., 2017; Guo et al., 2019; Wang et al., 2019; Wang et al., 2018; Yu et al., 2011; Yu et al., 2008a; Yu et al., 2008b). Experimental data from our study indicated that TOB1 may induce autophagy in gastric cancer cells.

We transiently overexpressed TOB1 in gastric cancer AGS cells and performed immunoblotting to determine the effect on LC3 protein. The autophagy molecule LC3 is found in two forms in the cell: LC3-I and LC3-II. During autophagy, cytoplasmic LC3-I loses a small polypeptide and then is conjugated to phosphatidylethanolamine, thereby generating LC3-II, which is localized to the inner and outer membranes of autophagosomes. The formation of LC3-II depends on ATG3 and ATG7. Unlike other Atg proteins that are inconsistently located on the membrane of autophagosomes, LC3-II is always localized on the membrane of autophagosomes until they fuse with lysosomes, making it an ideal molecular marker of autophagosomes (Kabeya et al., 2000; Kabeya et al., 2004; Pankiv et al., 2007; Tanida et al., 2003). Therefore, the level of autophagy can be estimated by measuring the conversion of LC3-I to LC3-II. We found that the conversion of LC3-I to LC3-II was significantly increased in gastric cancer cells 48 h after overexpressing TOB1. The increase in the LC3-II/LC3-I ratio reflected the potentiation of autophagy, which was maintained for 72 h after overexpressing TOB1 in gastric cancer cells. This result was confirmed by the typical autophagosome appearance under a transmission electron microscope. Moreover, correlation analysis showed that TOB1 was positively correlated with the expression of ATG3 and ATG7 in gastric cancer but not in normal gastric tissue. These findings indicated that the *TOB1* gene may induce autophagy in gastric cancer cells.

We then used a dual fluorescent GFP-RFP-LC3 fusion protein and a fluorescence microscope to trace autophagy in AGS cells overexpressing TOB1. GFP-RFP-LC3 adenovirus can be used to track the dynamic process of autophagy by labeling LC3 with GFP and RFP. In the absence of autophagy, red and green fluorescence appears uniformly distributed in gastric cancer cells, but when autophagy occurs, LC3 puncta formed by the aggregation of GFP and RFP on autophagosomes can be observed by laser confocal microscopy (Nie et al., 2016). We revealed that LC3 puncta formation in gastric cancer cells could be induced and further potentiated following TOB1 overexpression, indicating that the *TOB1* gene can induce autophagy in gastric tumor cells.

Finally, we observed autophagosomes, structural markers of autophagy, by transmission electron microscopy in AGS cells overexpressing TOB1. Autophagosomes are usually bilayer membrane structures containing cytoplasmic components or organelles (Ericsson, 1969; Nixon et al., 2005). The morphology and electron density of autophagosomes in the cytoplasm are consistent upon observation by electron microscopy, so they are easy to identify. After autophagosomes fuse to generate autolysosomes, they adopt a monolayer membrane structure containing cytoplasmic components at different degradation stages (Swanlund, Kregel & Oberley, 2010). Through transmission electron microscopy, we observed that autophagosomes were more common in gastric cancer cells with TOB1 overexpression than in control cells. Our results provide direct evidence of TOB1-induced autophagy in gastric cancer cells.

In addition, western blot data revealed that high TOB1 protein levels were associated with the formation of autophagosomes by suppressing the phosphorylation activation of AKT and mTOR in gastric cancer cells. The AKT/mTOR pathway is frequently activated in various tumor types (including gastric cancer) (Tapia et al., 2014; Tasioudi et al., 2015). TOB1 can inhibit the AKT/mTOR pathway in multiple cancers (Jiao et al., 2012; O’Malley et al., 2009). It is well known that the mTOR pathway functions as an autophagy regulator under starvation or other cellular stress conditions (Guertin & Sabatini, 2007). Since mTOR is a major downstream target of AKT, inhibition of the AKT/mTOR pathway could possibly trigger autophagy in tumor cells (Degtyarev et al., 2008).

These data indicated that the phosphorylation inactivation of the AKT/mTOR signaling pathway is critical for TOB1-induced autophagy in gastric cancer. In our data, AGS cells overexpressing TOB1 had significantly decreased levels of p-AKT (Ser473) and p-mTOR (Ser2448), suggesting that decreasing the phosphorylation of AKT at Ser473 and of mTOR at Ser2448 may play a crucial role in TOB1-induced autophagy. Previous reports suggested contradictory implications of AKT or mTOR phosphorylation. Phosphorylation of AKT at Ser473 is a novel biomarker to identify patients with advanced head and neck squamous cell carcinoma (HNSCC) at high risk for treatment failure following radiotherapy, and data generated using *ex vivo* tissue cultures support the hypothesis that pharmacological inhibition of AKT phosphorylation at Ser473 might circumvent radioresistance to improve the efficacy and reduce the toxicity of current treatment modalities (Freudlsperger et al., 2015). Meanwhile, the phosphorylation of mTOR at Ser2448 and Ser2481 markedly suppressed the survival of hepatocellular carcinoma (HCC) cells (Watari et al., 2016). Therefore, the mechanism by which inactivation (no phosphorylation) of the AKT (Ser473)/mTOR (Ser2448) pathway affects TOB1-induced autophagy in gastric tumor cells needs to be further explored.

## Conclusion

Altogether, the present results strongly support our hypothesis that TOB1 promotes autophagy in gastric cancer cells. Furthermore, TOB1-induced autophagy might inhibit the growth of AGS cells (functioning as protective autophagy that antagonizes gastric cancer) by inhibiting the phosphorylation of AKT/mTOR; these findings are consistent with our previous data showing the antiproliferative activity of TOB1 in AGS cells (Guan et al., 2017). Further evidence is required to confirm the relationship between autophagy and the anticancer activity of TOB1 *in vitro* and *in vivo*, as well as to validate this relationship in clinical settings. Clarification of these remaining unknowns will provide a reliable foundation for using TOB1 as a biomarker to improve therapeutic efficacy in patients with gastric cancer.

## Supplemental Information

10.7717/peerj.12904/supp-1 Supplemental Information 1 Raw D. Uncropped scans of membranes used for Western blot images

10.7717/peerj.12904/supp-2 Supplemental Information 2 The raw numbers for the Figures.

## Additional Information and Declarations

### Data Availability

The following information was supplied regarding data availability: The raw data are available in the Supplemental File.
