# Supplementary material for: Involvement of TOB1 on autophagy in gastric cancer AGS cells via decreasing the activation of AKT/mTOR signaling pathway
Source: PeerJ. 2022 Feb 4;10:e12904. doi: 10.7717/peerj.12904 (PMC8820212; doi:10.7717/peerj.12904)

**Figure1.** (n=3)


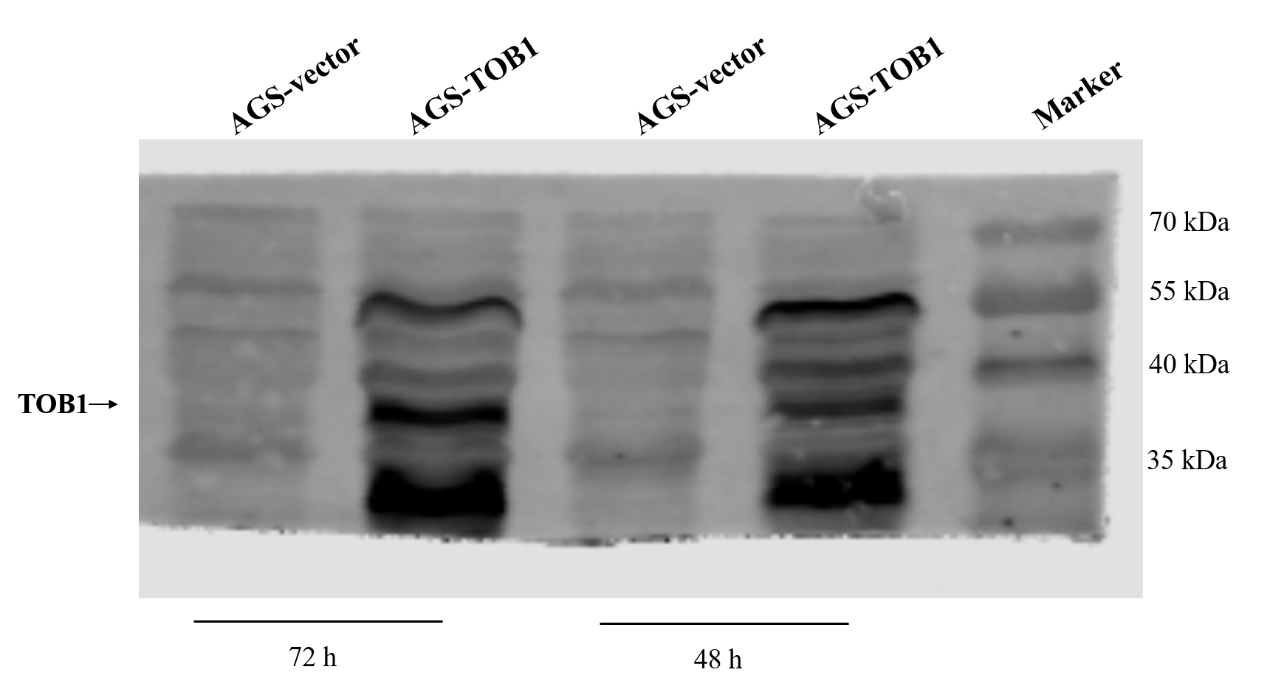


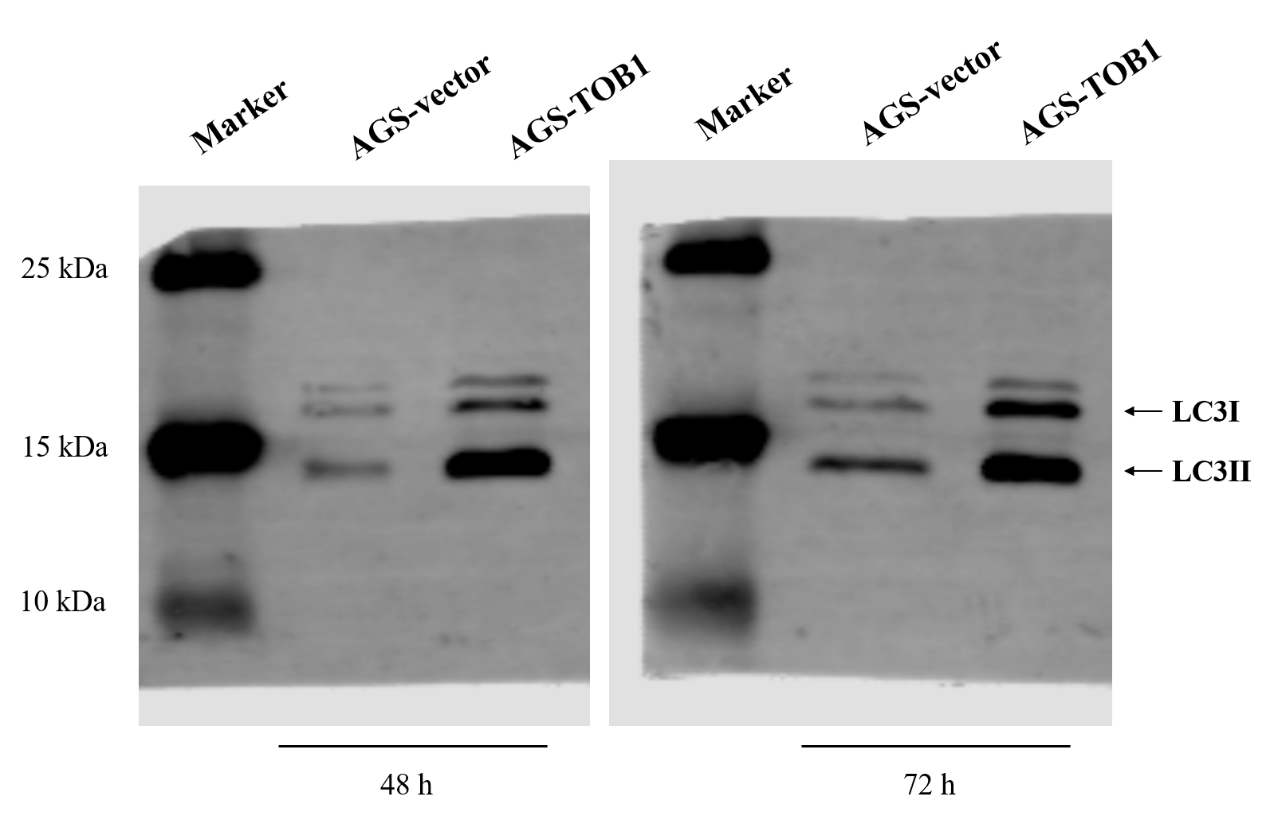


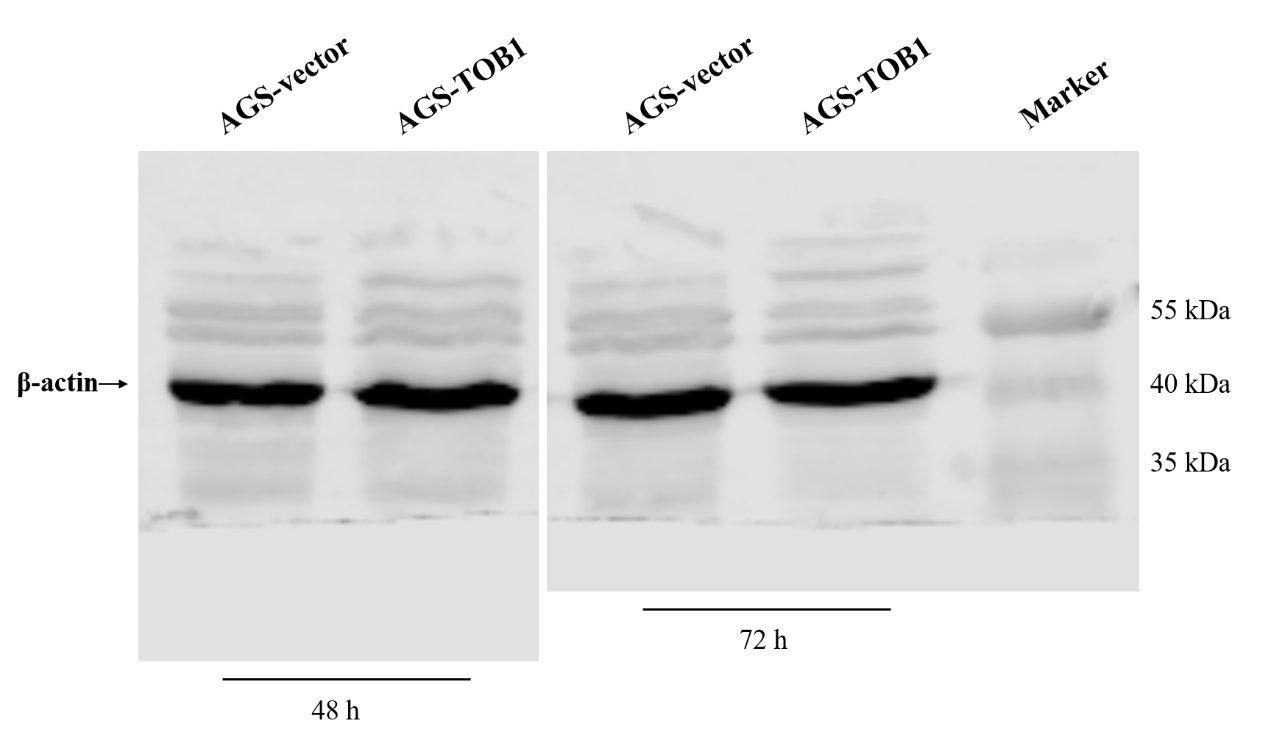


Repeating data for TOB1


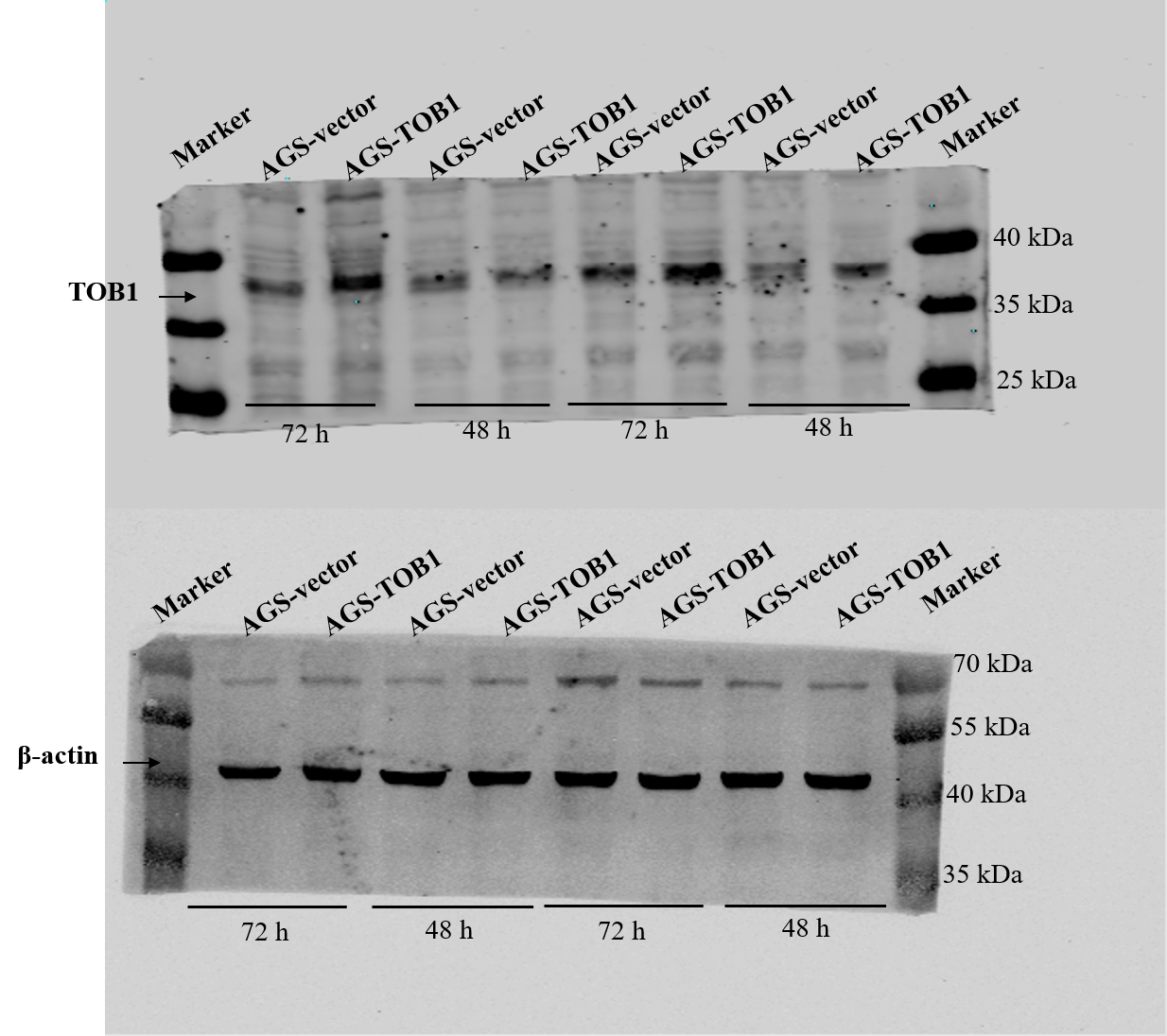


Repeating data for LC3


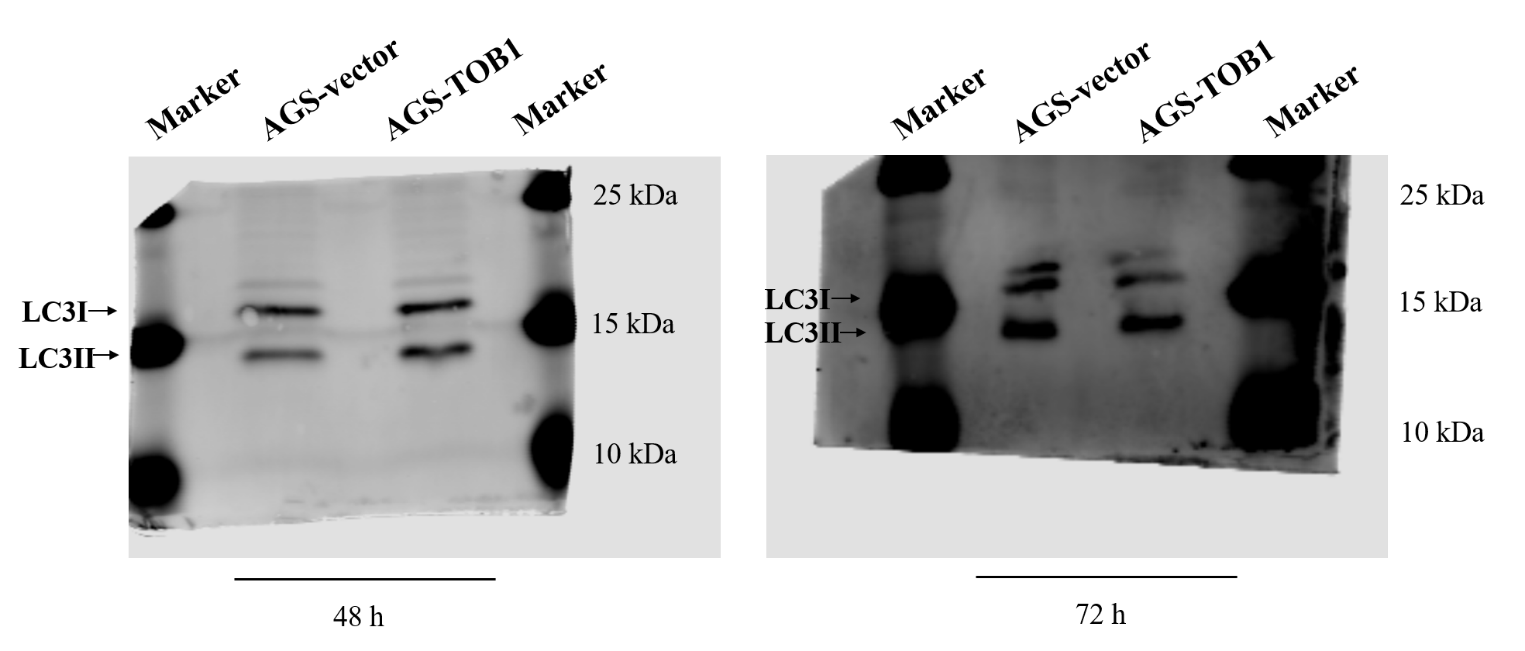


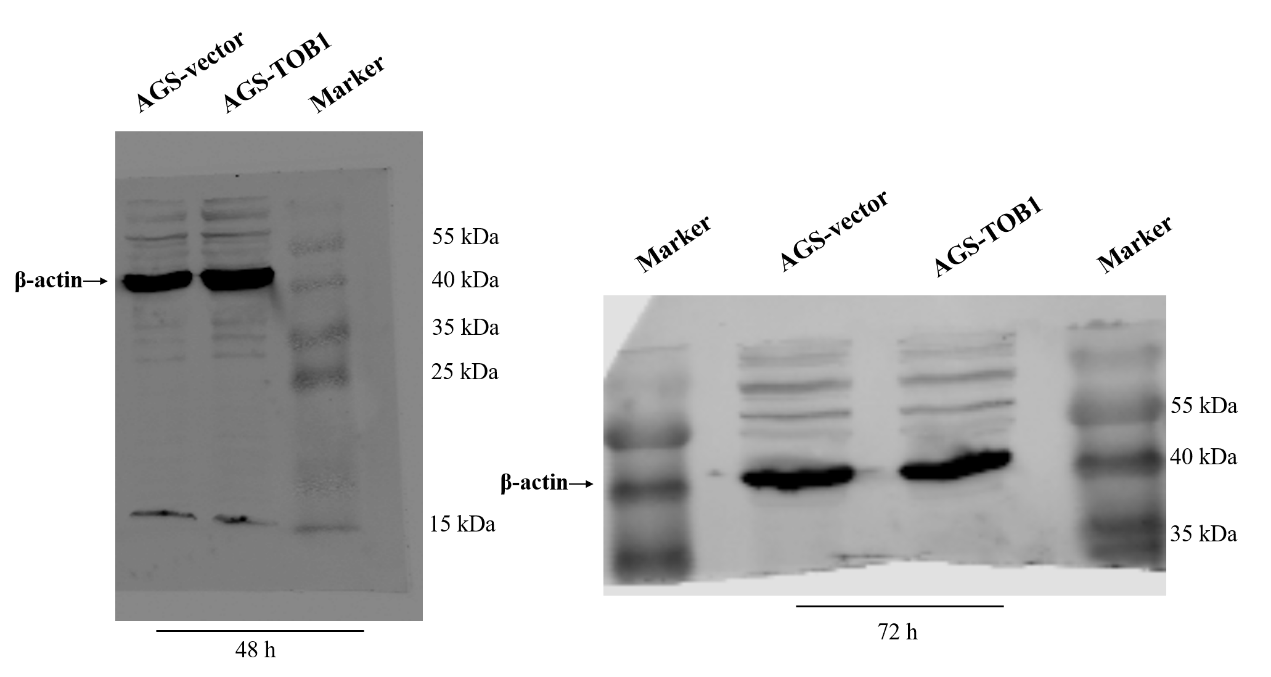


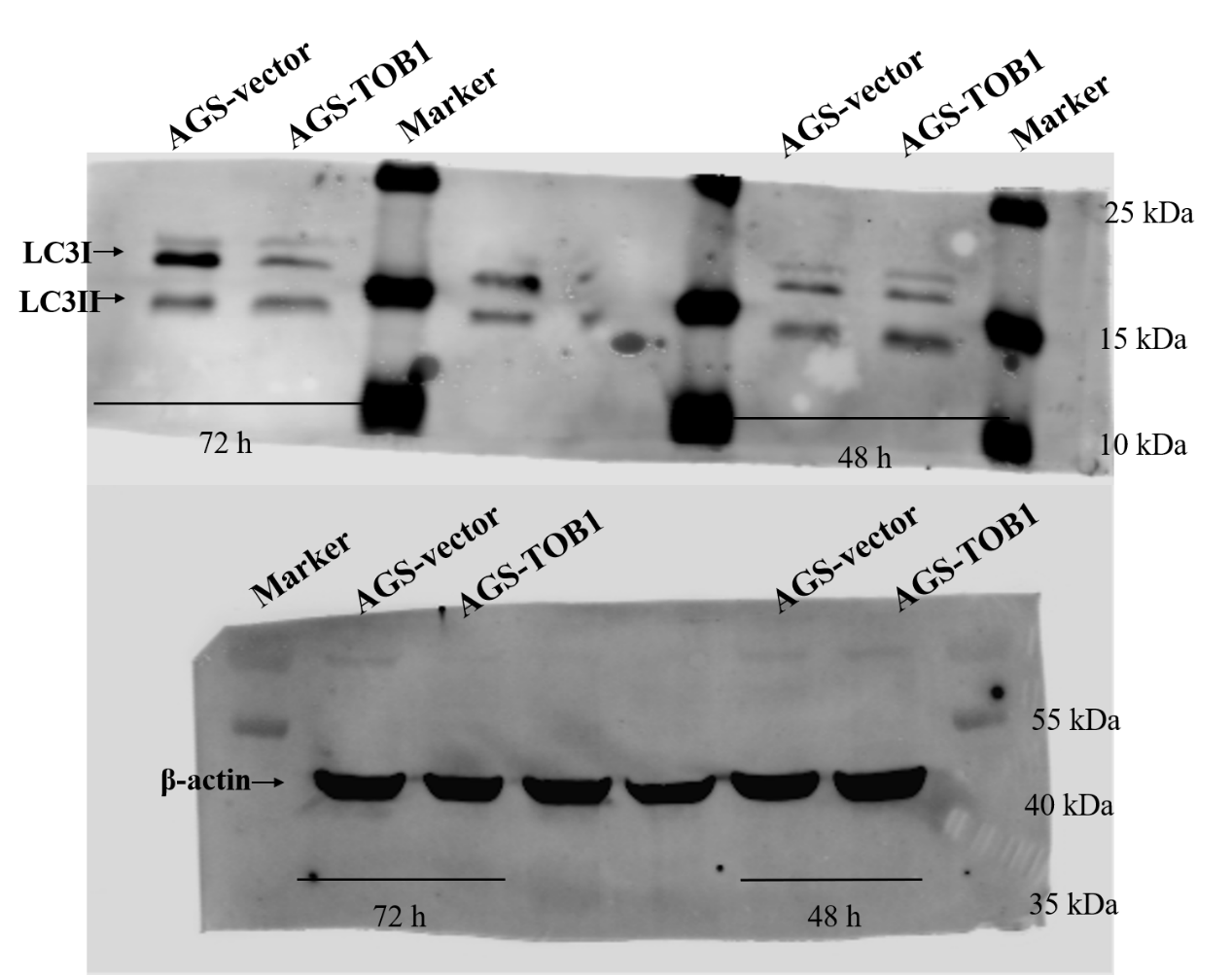


**Figure5.** (n=3)


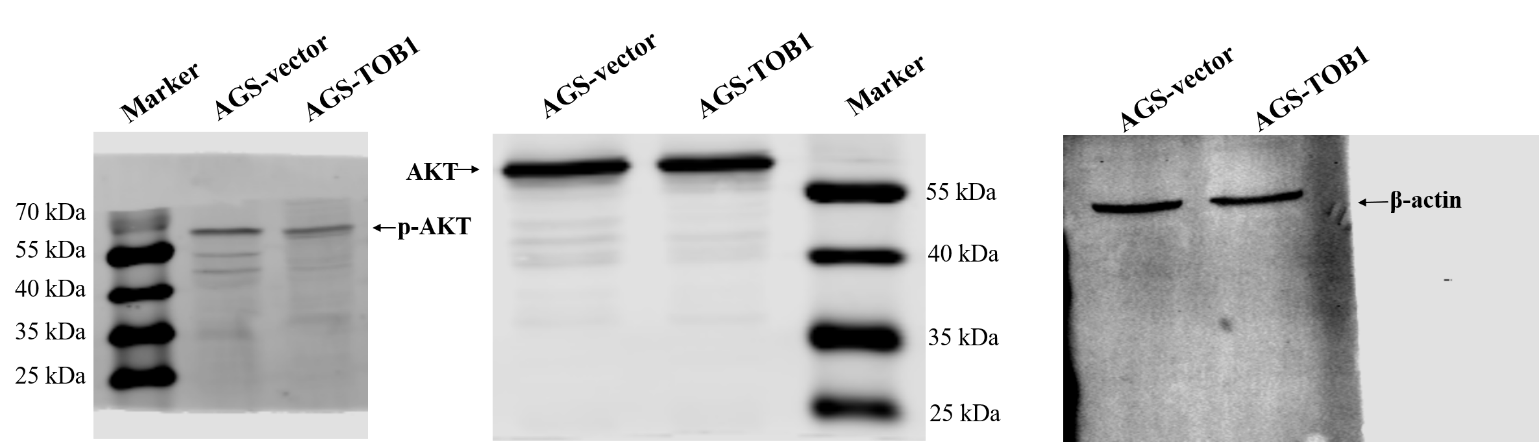


Repeating data for p-AKT and AKT


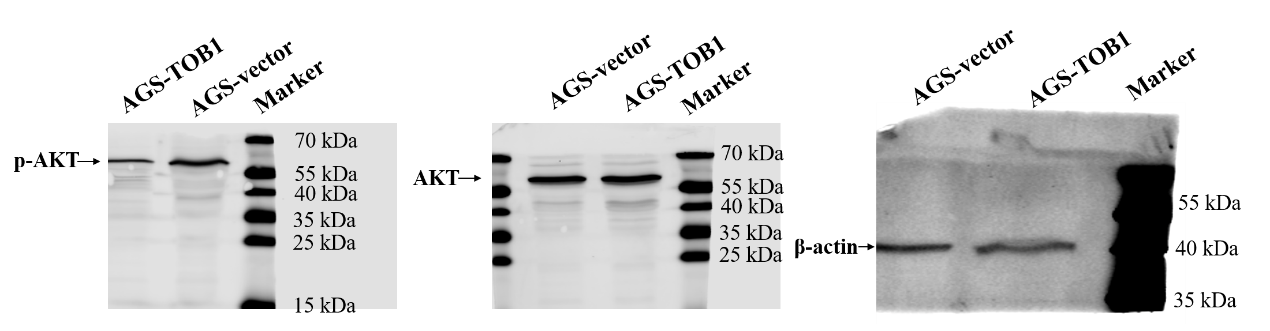


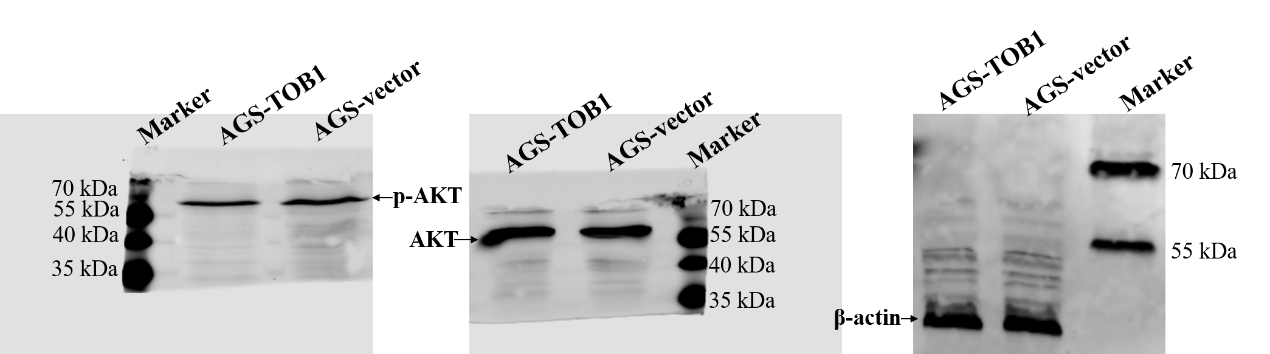


**Figure5.** (n=2)


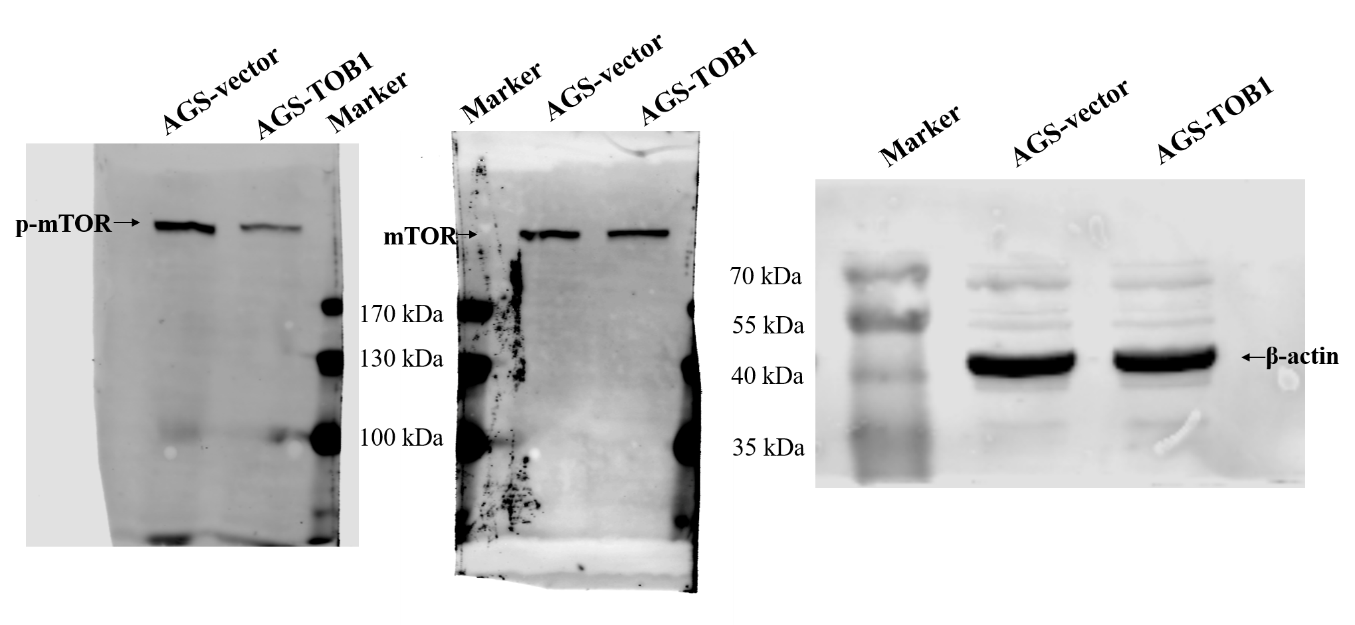


Repeating data for p-mTOR and mTOR


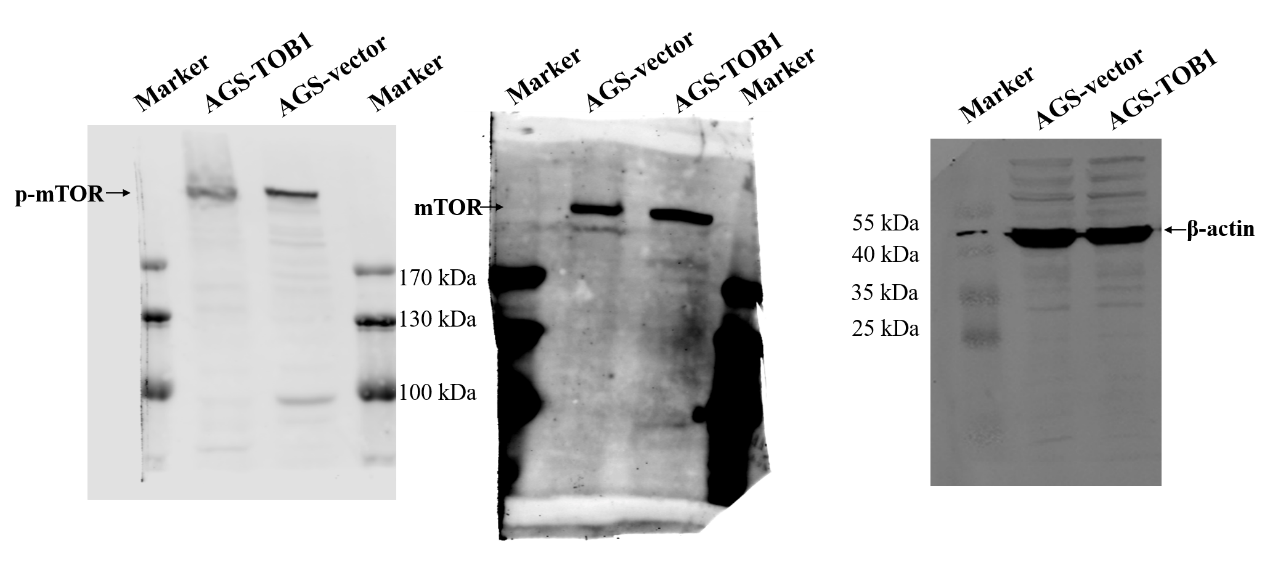

Supplement: Supplemental Information 1 — Uncropped scans of membranes used for Western blot images [file peerj-10-12904-s001.docx]
